# Bilirubin Hepatic and Intestinal Transport and Catabolism: Physiology, Pathophysiology, and Benefits

**DOI:** 10.3390/antiox14111326

**Published:** 2025-11-03

**Authors:** Zachary A. Kipp, Sally N. Pauss, Genesee J. Martinez, Terry D. Hinds, Wang-Hsin Lee

**Affiliations:** 1 Drug & Disease Discovery D3 Research Center, Department of Pharmacology and Nutritional Sciences, University of Kentucky College of Medicine, Lexington, KY 40508, USA; zachary.kipp@uky.edu (Z.A.K.); sally.pauss@uky.edu (S.N.P.); genesee.martinez@uky.edu (G.J.M.); 2 Barnstable Brown Diabetes Center, University of Kentucky College of Medicine, Lexington, KY 40536, USA; 3 Markey Cancer Center, University of Kentucky, Lexington, KY 40536, USA

**Keywords:** liver disease, obesity, MASLD, NAFLD, cardiovascular disease, gut microbiota, HMOX, HO-1, BVRA, UGT1A1

## Abstract

Bilirubin, a metabolite derived from heme degradation, has traditionally been regarded as a waste product and a marker of liver injury. However, increasing evidence suggests that bilirubin also functions as a hormone, and reduced levels are associated with metabolic dysfunction. Studies have shown a strong association between low circulating bilirubin levels and an increased risk of metabolic disorders and cardiovascular disease. To advance bilirubin-based treatment strategies, it is essential to elucidate the mechanisms underlying bilirubin transport and metabolism. Therefore, we provide an in-depth discussion of bilirubin production and its subsequent fates, with a particular focus on the transport between the liver and the intestine. We describe the molecular players involved in heme degradation and biliverdin formation, leading to bilirubin production, followed by its transport from the bloodstream to hepatocytes and from the liver to the intestine. We discuss intestinal bilirubin catabolism, including the microbiome generation of urobilinogen, urobilin, and other metabolites. Finally, we discuss how bilirubin clearance and catabolism intersect with its metabolic effects, highlighting potential therapeutic targets. By integrating these aspects, this review provides a comprehensive understanding of bilirubin’s physiological importance, intestinal transport, and breakdown, as well as insights into novel strategies for treating hypobilirubinemia-associated disorders.

## 1. Introduction

Bilirubin, a product of heme degradation, has long been regarded as a toxic compound readily cleared from the human body. However, emerging evidence has confirmed its hormonal functions by binding to its endogenous receptor, PPARα (peroxisome proliferator-activated receptor alpha) [1,2,3,4,5]. Interestingly, bilirubin metabolites may exert opposing effects, contributing to the development of metabolic dysfunction and disease [6]. To better understand bilirubin and its derivatives, it is essential to detail the mechanisms of bilirubin transport and catabolism. Therefore, this review aims to explore the mechanisms underlying bilirubin’s transport and breakdown in the human body, particularly in the liver and intestines.

The primary site of bilirubin breakdown is the liver hepatocytes [7], which are crucial for converting hydrophobic, unconjugated bilirubin into a more water-soluble conjugated form. This process relies on the enzyme uridine diphosphoglucuronate-glucuronosyltransferase 1A1 (UGT1A1) [8]. The movement of unconjugated bilirubin from the bloodstream involves its import into hepatocytes and conjugation with glucuronic acid by UGT1A1. The subsequent excretion of conjugated bilirubin via bile depends on several transporters, including ATP-binding cassette subfamily C member 2 (ABCC2/MRP2), as well as anion transporting polypeptides 1 B1 (OATP1B1) and 1 B3 (OATP1B3). Malfunctions in these enzymes or transporters can lead to mild or extreme increases in plasma bilirubin levels, contributing to conditions such as Gilbert Syndrome, Crigler–Najjar Syndrome, Dubin–Johnson Syndrome, and Rotor Syndrome. Gilbert Syndrome is caused by a homozygous polymorphism in the *UGT1A1* gene promoter [2], leading to decreased hepatic glucuronidation and bilirubin excretion [9]. Similarly, Crigler–Najjar Syndrome results from mutations that suppress liver UGT1A1 activity, with type 1 leading to complete loss and type 2 causing reduced enzyme activity, resulting in decreased hepatic glucuronidation of bilirubin and a buildup of unconjugated bilirubin in the circulation [10,11]. Reduced UGT1A1 enzyme activity is not the only factor that can alter bilirubin transport and breakdown. Dubin–Johnson Syndrome is characterized by impaired excretion of conjugated bilirubin due to a mutation in the ABCC2/MRP2 gene [12]. Rotor Syndrome, on the other hand, involves deficiencies in OATP1B1 and OATP1B3, which reduce the liver’s ability to take up bilirubin and raise circulating levels [13].

This review also examines how these essential components collaborate to regulate bilirubin metabolism. The bilirubin metabolic process does not terminate upon its excretion into the intestinal lumen. Once conjugated, bilirubin arrives in the intestine and the gut microbiota transforms it into various derivatives, including urobilinogen, urobilin, and other metabolites [7,14]. Some of these substances may be reabsorbed into the bloodstream; however, their physiological roles remain inadequately understood.

Bilirubin buildup in cases of extreme hyperbilirubinemia (>150 μM) causes jaundice [2], which is used clinically as an indicator of liver damage or bile duct blockage. This likely occurs because hepatocytes die, and the liver is unable to conjugate bilirubin for removal from the circulation via hepatic UGT1A1. However, bilirubin accumulation is not always harmful to human health. A mild increase in plasma bilirubin (25–58 μM), like that seen in Gilbert’s syndrome [2], has been linked to protective effects against the risks of heart disease [15,16,17,18,19,20]. Other research has also supported bilirubin’s role as a hormone and its potential health benefits in the treatment of metabolic disorders [21,22,23,24,25,26,27,28]. Consequently, we also explore the possible roles of bilirubin-derived metabolites in metabolic disease. These studies suggest that blocking bilirubin’s breakdown could be a promising new approach for treating various health issues. Therefore, this review examines the potential of targeting key enzymes and transporters involved in bilirubin homeostasis, and bilirubin nanoparticles and their therapeutic options.

## 2. Bilirubin Production and Heme Degradation

### 2.1. Heme Degradation Pathway

**Figure 1:**
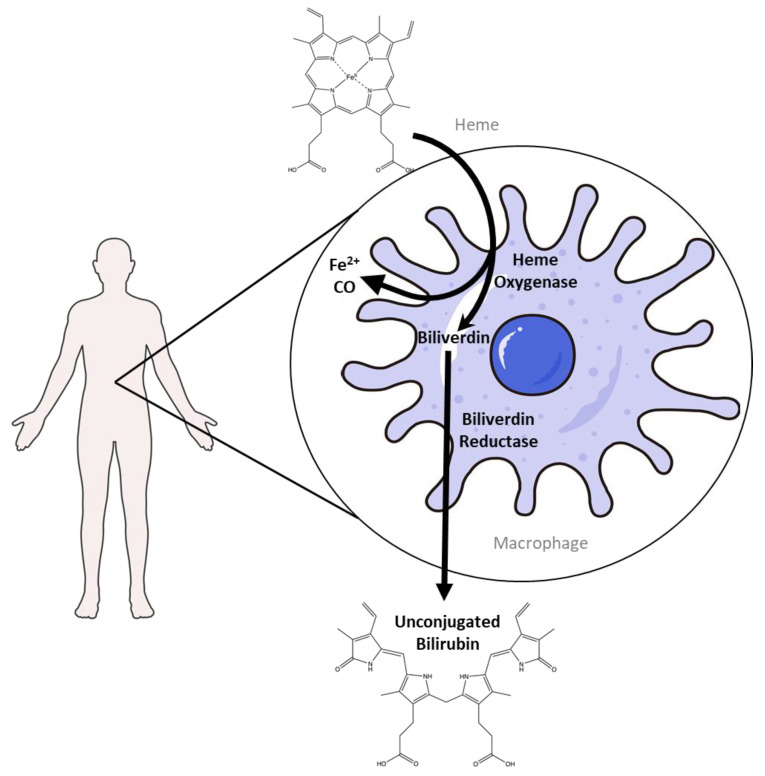
Heme catabolism occurs primarily in the spleen, where macrophages phagocytose red blood cells. Heme oxygenase is the rate-limiting enzyme that degrades heme. In this process, heme is converted into biliverdin, releasing carbon monoxide and ferrous iron. Biliverdin is then converted to unconjugated bilirubin by biliverdin reductase (Created in BioRender. Lee, W. (2025) https://BioRender.com/6of7a7t) The macrophage image was generated by adapting Adobe Stock photo #621007676 (macrophage) licensed to the University of Kentucky.

Bilirubin production starts with heme degradation by heme oxygenase (HO), which produces biliverdin, carbon monoxide, and ferrous iron. The process primarily occurs in the spleen, although not exclusively, as other tissues can also reduce biliverdin. This happens when effete red blood cells are phagocytized by macrophages and degraded in the phagolysosome (Figure 1) [29,30]. Degradation of heme by HO is the rate-limiting step of heme catabolism. Heme is considered a substrate and cofactor for its own degradation via HO [31]. Increases in hemoglobin increase the expression of HO [32]. There are two main forms of HO, HO-1 and HO-2 (gene names are *HMOX1* and *HMOX2*, respectively) [21]. HO-1 is the inducible version of heme oxygenase, while HO-2 is constitutively expressed [33]. Tissue distribution between the two is also different. HO-2 is predominantly found in the brain, testis, and carotid bodies [34,35], while HO-1 is highest in spleen, liver, and bone marrow [36,37].

HO-1 can be induced by various factors, including hypoxia, inflammation, and oxidative stress [38,39,40,41]. Both HO isozymes are important for cellular protection against oxidative stress and inflammation. HO-1 is regulated in a highly conserved manner by nuclear factor erythroid 2-related factor 2 (NRF2), a transcription factor that controls the expression of many anti-inflammatory and antioxidant proteins [42,43,44,45]. An increase in HO-1 has been shown to protect against ischemia injury, oxidative stress, inflammation, apoptosis, and cell proliferation [39,42,46,47,48,49]. When HO catalyzes cleavage of the heme ring, it occurs at the α-methene bridge carbon, forming biliverdin [50]. Biliverdin is converted to bilirubin by biliverdin reductase (BVR), a process discussed in the next subsection.

### 2.2. The Production of Bilirubin and Its Isoforms

**Figure 2:**
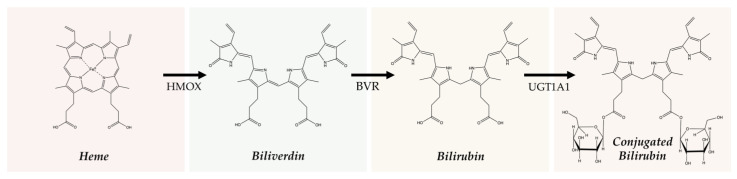
The structure changes from heme to conjugated bilirubin. HMOX cleaves the porphyrin ring of heme, forming biliverdin. BVR then reduces biliverdin to bilirubin. UGT1A1 then conjugates bilirubin with glucuronic acid in the liver.

When first produced, bilirubin is in its unconjugated form and is not water-soluble. Unconjugated bilirubin can travel through the bloodstream by binding to albumin [51,52]. The liver may then take up unconjugated bilirubin from the bloodstream via the organic anion-transporting polypeptides OATP1B1 and OATP1B3 [53]. In the liver, UGT1A1 conjugates bilirubin with glucuronic acid (Figure 2). This process allows bilirubin to become water-soluble, and the resulting compound is referred to as conjugated bilirubin. Bilirubin can then be excreted in the bile and transported to the small intestine via the biliary duct, where it is catabolized to urobilinogen and rapidly oxidized to urobilin (this process has been reviewed in [6,7]).

Biliverdin IXα is converted to bilirubin IXα through the enzymatic reaction by the BVRA isoform [54]. In humans, there is one other isoform, BVRB, that reduces biliverdin IXβ to bilirubin IXβ [55]. At nearly all times, bilirubin IXα is the predominant isoform, comprising about 97% of plasma bilirubin [56] (Figure 2). However, up to 22 weeks of gestation, bilirubin IXβ was found to be the predominant form in fetal bile samples [56]. Bilirubin IXβ is hydrophilic and water-soluble, unlike bilirubin IXα, and therefore may be excreted without conjugation and have less antioxidant abilities [56]. Bilirubin IXα is the form present in adults, and it is produced primarily (80%) from the breakdown of hemoglobin, but it can also be made (20%) from the breakdown of other hemoproteins [57]. The major isoform of bilirubin is bilirubin IXα, and other bilirubin forms that also exist are displayed in Table 1. Most of the other bilirubin molecules are present in very small amounts in the bloodstream [14,58]. There is limited data available on bilirubin III and bilirubin XIII, as they are not commonly found or produced in the body; however, they have been identified in standard reference materials [59].

**Figure 3:**
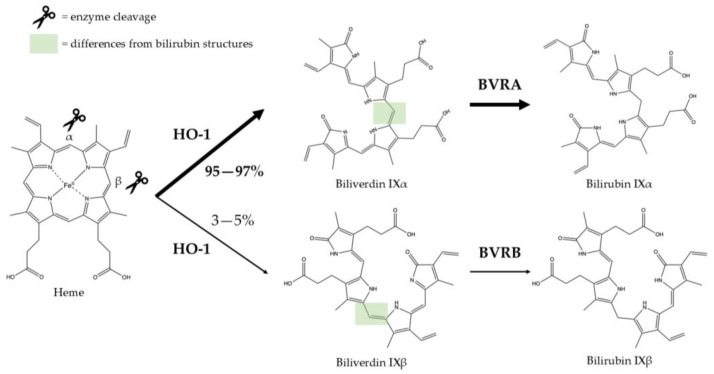
Production of bilirubin IXα and IXβ. Heme oxygenase 1 (HO-1) cleaves heme at the alpha bridge to produce biliverdin Ixα, and this accounts for about 95% of biliverdin in humans. Alternatively, HO-1 cleaves the β bridge to produce biliverdin IXβ. Biliverdin IXα and IXβ are further metabolized by biliverdin reductase A and B (BVRA and BVRB), respectively. This results in the formation of bilirubin IXα and bilirubin IXβ.

Bilirubin has a tetrapyrrolic structure, which is a result of the breakdown of the protoporphyrin (heme) ring. Heme is composed of protoporphyrin IX and Fe^2+^. HO catalyzes the reaction of heme to biliverdin by using oxygen and electrons to cleave the porphyrin ring of heme into biliverdin, which is a linear molecule made up of four pyrrolic rings, as well as carbon monoxide and iron. BVR then utilizes NADPH to reduce biliverdin to bilirubin. The bilirubin IXα is formed by the stereospecific cleavage of the α-methene bridge of protohaem-IX [59] (Figure 3). In neonatal jaundice, newborns experience high levels of bilirubin in the blood; the condition can be mild or severe and is usually treated to prevent brain damage if levels are too high. When neonatal jaundice is treated with phototherapy, bilirubin IXα photoisoforms *ZE*, *EZ*, and *EE* (Table 1) are formed [60]. These isoforms are more hydrophilic and thus can be excreted in the bile without the need for conjugation [60].

Multiple modifiable factors may control plasma bilirubin levels, including exercise and diet. High-running capacity rats have elevated hepatic BVRA and lower UGT1A1, leading to significantly higher plasma bilirubin levels than in low-running capacity rats [62]. In humans, moderate to severe exercise (an average of 169 min per week) increased plasma bilirubin levels [63]. Although human data linking exercise and bilirubin is limited, their link and potential implications have been reviewed in [64]. For patients with Gilbert’s syndrome, one study recommended avoiding excessive calorie restriction (<400 calories/day) and certain vegetables and fruits (in the *Cruciferae, Apiaceous, Rutaceae* groups) to prevent jaundice episodes, indicating that these nutritional interventions increase plasma bilirubin [65]. In a study of over 3000 participants, plasma urobilin was the only metabolite that decreased in response to a healthy, conscious dietary pattern [66]. These studies indicate the importance of dietary intake and exercise in regulating plasma bilirubin and urobilin levels, but further studies are needed to determine the direct mechanisms.

## 3. Hepatic Bilirubin Excretion

**Figure 4:**
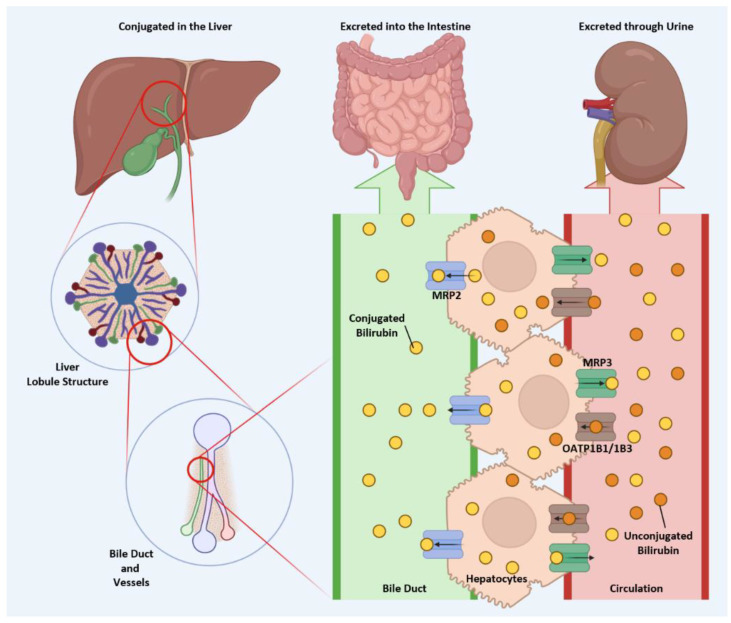
MRP2 and MRP3 determine the metabolic fate of conjugated bilirubin. Unconjugated bilirubin enters hepatocytes through OATP1B1 and OATP1B3. After conjugation in the liver, bilirubin can be transported into the bile duct by MRP2 or released into circulation via MRP3. The conjugated bilirubin in the bile duct enters the intestine, where it interacts with the gut microbiota and undergoes microbial metabolism. In contrast, conjugated bilirubin entering the circulation is excreted in the urine. (Created in BioRender. Lee, W. (2025) https://BioRender.com/9qccjmy).

Once bilirubin is conjugated in the liver, it is ready to exit hepatocytes and begin its next phase of transport. Multidrug resistance protein 2 (MRP2) and 3 (MRP3) mediate this process by pumping conjugated bilirubin into the bile duct and bloodstream, respectively (Figure 4). These two pathways have distinct outcomes: conjugated bilirubin, secreted into the bile duct, travels with bile salts to the small intestine, whereas conjugated bilirubin released into the bloodstream is ultimately excreted in the urine. MRP2, a key transporter responsible for biliary excretion of conjugated bilirubin, is located on the canalicular membrane of hepatocytes. It pumps organic anions, bile salts, and drug metabolites from hepatocytes into bile [67]. Regulation of MRP2 activity, therefore, directly affects bilirubin clearance. For instance, activation of the PI3K/Akt signaling pathway suppresses MRP2 availability on the hepatocyte surface [68], thereby reducing the efficiency of bilirubin excretion. Similarly, primary biliary cirrhosis is associated with decreased MRP2 expression [67], leading to impaired clearance of conjugated bilirubin. The potential of targeting MRP2 in therapeutic strategies will be discussed later.

## 4. Gut Microbiome Metabolism of Bilirubin

Bilirubin is conjugated in the liver, making it more water-soluble and allowing it to be secreted into bile, which is then excreted into the intestines via the biliary tract. Conjugated bilirubin is deconjugated by β-glucuronidases (GUS), which are present in human cells and gut microbiota within the intestine [4]. GUS enzymes are highly conserved and are found across all four main bacterial phyla in the human gut (Table 2) [64]. Proteolytic activity is tightly regulated to preserve the integrity of the intestinal barrier. In irritable bowel syndrome (IBS), patients may experience increased protease activity, potentially causing injury [65]. Patients with high proteolytic activity IBS have lower levels of urobilinogen and decreased GUS activity [65]. Administration of unconjugated bilirubin and increased GUS activity inhibit serine proteases, reducing proteolytic activity [65]. This underscores the role of unconjugated bilirubin in supporting gut health and suggests therapeutic potential for IBS.

**Figure 5:**
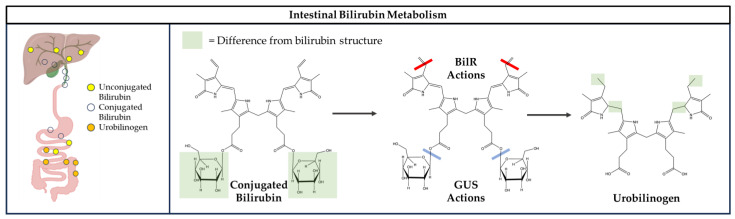
Bacterial enzymes facilitate the conversion of conjugated bilirubin into urobilinogen. Once within the liver, bilirubin undergoes conjugation and is transported via the biliary tract into the intestine. In the intestinal environment, urobilinogen may undergo further metabolic processes. Conjugated bilirubin’s double bonds are reduced by bilirubin reductase (BilR), while β-glucuronidases (GUS) remove the glucuronic acid molecules, resulting in the formation of urobilinogen. Urobilinogen differs from bilirubin in that it contains single bonds, as illustrated by the green boxes, which emphasize the structural variation from bilirubin. (Created in BioRender. Pauss, S. (2025) https://BioRender.com/m1a62ii).

Previously, it was thought that the conversion of bilirubin to urobilinogen may need multiple enzymes. However, Hall et al. demonstrated that a single enzyme, bilirubin reductase (BilR), controlled the process [69]. BilR is an enzyme of gut microbial origin that metabolizes bilirubin [6]. BilR performs double-bond reductions in bilirubin (Figure 5) [70]. Because conjugation and deconjugation of bilirubin are reversible processes, it is suspected that BilR may be the determinant of bilirubin reabsorption or excretion [69].

Many of the identified bilirubin-reducing bacteria belong to the Clostridium genus within the Firmicutes phylum (Table 2) [69]. The BilR enzyme was detected in nearly all individuals, though absent in many infants; it was generally present by the age of one year, which may suggest a potential role in neonatal jaundice, a condition characterized by the accumulation of unconjugated bilirubin [69]. Considering that patients with inflammatory bowel disease (IBD) exhibit lower serum bilirubin levels, a higher proportion of this population lacks the BilR enzyme, prevalence exceeding 30%, compared to 0.1% in the general population [69].

Given the clinical relevance of the BilR and GUS bacterial enzymes in the intestine (Table 3), it is warranted to consider how antibiotics may affect bilirubin levels. It has been demonstrated that rats colonized with bilirubin-reducing bacteria have lower serum bilirubin levels than those colonized with non-reducing bacteria [71]. As suggested by Vitek and Tiribelli, serum bilirubin—a measurement sometimes used to assess liver damage—may sometimes instead reflect gut microbiota activity [70].

Urobilinogen shares similarities with bilirubin and may act as an antioxidant in the large intestine (Table 3). When evaluating antioxidant capacity using DPPH (2,2-diphenyl-1-picrylhydrazyl) radical scavenging, urobilinogen outperformed bilirubin, α-tocopherol, and β-carotene [72]. This suggests that in the large intestine, where bilirubin levels are very low due to rapid conversion, urobilinogen might serve as a more effective antioxidant. Urobilinogen is readily oxidized to urobilin in the intestine or kidneys [73], a water-soluble compound that can be reabsorbed through the hepatic portal system [6]. Urobilin is responsible for the yellow color of urine and may function as a urine biomarker for heart attacks (discussed further in [6]). Unlike bilirubin and urobilinogen, urobilin has no known antioxidant properties. Plasma bilirubin levels have been associated with protection of cardiovascular tissues and prevention of stroke [6,20,74,75]. However, urobilin is positively correlated with cardiometabolic diseases and has been proposed as a urine biomarker [6].

Following the conversion of bilirubin to urobilinogen and oxidation to urobilin, it may be absorbed within the intestines to enter circulation or undergo further breakdown by unidentified enzymes into stercobilin, which is subsequently excreted via feces [12]. Stercobilin is a dark orange pigment responsible for the characteristic coloration of feces [69]. Consequently, stercobilin and urobilinogen are utilized as indicators of fecal contamination in drinking water [70,71].

## 5. Intestinal Reabsorption of Bilirubin and Its Metabolites

**Figure 6:**
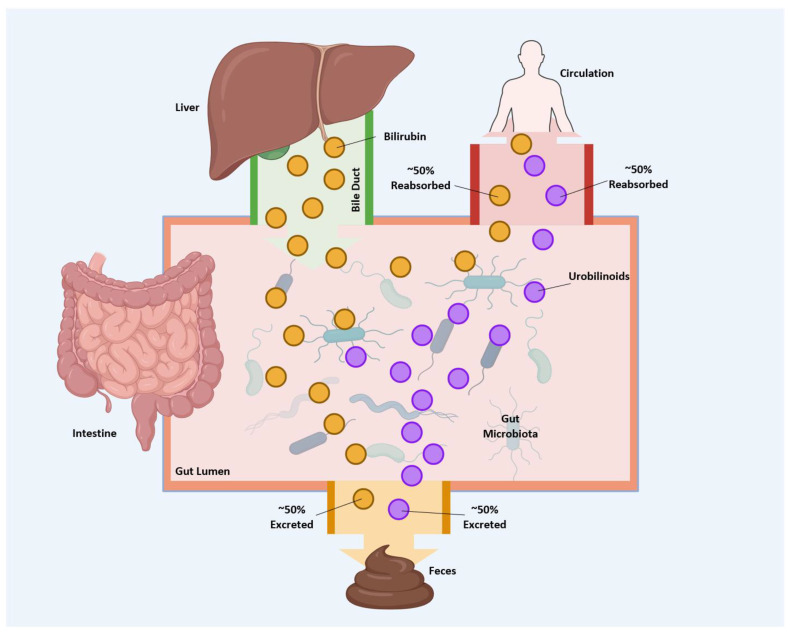
Reabsorption of bilirubin and urobilinoids. The liver secretes bilirubin into the biliary system, which is subsequently secreted into the intestine. Once bilirubin enters the intestinal lumen, gut microbiota converts a portion of it into urobilinoids. Approximately 50% of the bilirubin secreted by the liver is reabsorbed, while roughly half of the urobilinoids produced in the intestine are also reabsorbed. The remaining fractions of both bilirubin and urobilinoids are excreted via feces. (Created in BioRender. Lee, W. (2025) https://BioRender.com/rvfmwi0).

Approximately 50% of urobilin is reabsorbed through enterohepatic circulation, with the remaining excreted in feces [76] (Figure 6). However, in certain diseases, the amount of urobilin absorbed may be higher, as reviewed by Kipp et al. [6]. Conjugated bilirubin, a soluble and polar molecule, is expelled into the intestine via the biliary system [7].

Its chemical properties and larger size, compared to unconjugated bilirubin, limit its reuptake by enterocytes. After gut microbiota deconjugate bilirubin, it can be reabsorbed into the enterohepatic circulation. Studies in rats using radiolabeled bilirubin show that unconjugated bilirubin is rapidly absorbed from the intestines into the bloodstream [77]. Because it is lipid-soluble, unconjugated bilirubin can pass through the enterocyte plasma membrane by passive diffusion, without the need for a transporter [7]. While existing evidence supports passive diffusion as the primary mechanism for bilirubin entry into enterocytes, further research is needed to determine whether transporter-mediated uptake also occurs.

At present, little is understood about the mechanism of urobilin absorption. It is estimated that fifty percent of urobilinoids generated are reabsorbed into the enterohepatic circulation, whereas the remaining fifty percent are excreted via feces [76] (see Figure 6). Lester and Schmid demonstrated that urobilinogen can be absorbed along the entire length of the digestive tract [78]. In rats, following the absorption of urobilinogen, approximately ninety percent is excreted back into the intestines through the biliary system. In comparison, the remaining five to ten percent is excreted in the urine [79]. Although urobilin lacks a known physiological function, it has been shown to bind to albumin, which may serve as a carrier protein facilitating its transport from the liver to the kidneys [51]. Further research is warranted to elucidate whether transporters are essential for the intestinal absorption of urobilin.

Inhibition of intestinal urobilin absorption could represent a potential therapeutic approach for diseases characterized by elevated urobilin levels, including obesity, diabetes, and cardiovascular disorders [6]. In human subjects, plasma urobilin levels have been positively associated with markers of adiposity and insulin resistance, with a stronger correlation observed in females than in males [23]. Plasma urobilin has exhibited positive correlations with increased visceral fat area, oxidized low-density lipoprotein (LDL), LDL-cholesterol, and both systolic and diastolic blood pressure within a human cohort [80]. These findings have been corroborated by another study, which identified a positive association between plasma urobilin and significantly higher triglycerides, blood glucose, and BMI, and this was associated with all-cause mortality among over 700 diabetic patients [81]. Additionally, in three community-based cohorts, urobilin was linked to an increased incidence of heart failure and showed an inverse relationship with left ventricular ejection fraction [82]. Although multiple investigations have established an adverse association between urobilin and cardiometabolic diseases, further research is necessary to elucidate the mechanisms by which urobilin contributes to disease pathophysiology.

## 6. Physiological Consequences of Bilirubin Catabolism and Transport

**Figure 7:**
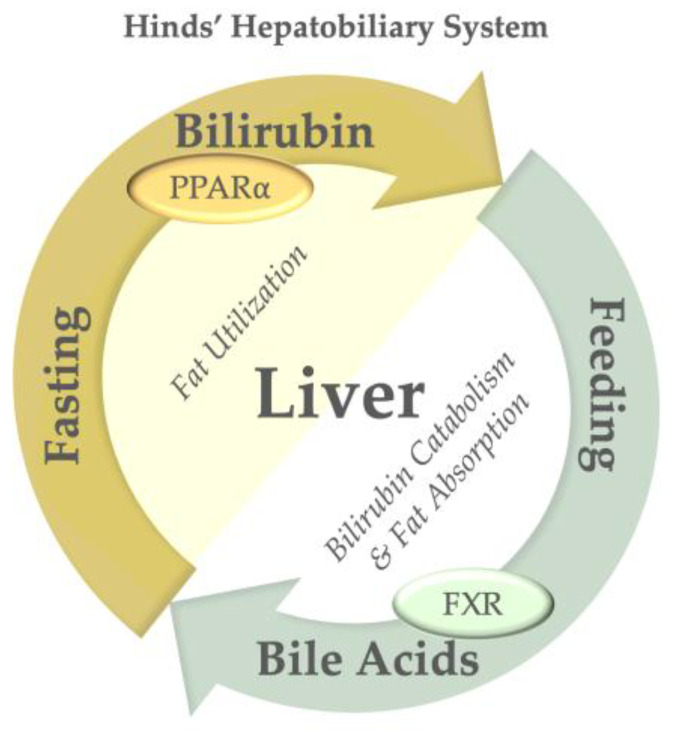
Hinds’ Hepatobiliary System and Fasting and Feeding Responses. Fasting increases PPARα expression and plasma bilirubin levels, thereby collectively stimulating fat utilization. Conversely, feeding increases hepatic bile acid production, activating hepatic and intestinal FXR and enhancing fat absorption in the gastrointestinal tract.

Research conducted by Barrett and colleagues as early as 1971 demonstrated that plasma bilirubin levels are significantly elevated during fasting periods in both humans and rodents [83,84,85]. During such fasting intervals, plasma bilirubin concentrations are modestly increased (3–4-fold) and then decline during feeding. Lee et al. postulated that this phenomenon results from bilirubin’s induction of lipid oxidation genes via its receptor, PPARα (see Figure 7), which is markedly upregulated during fasting [86]. Conversely, during feeding, the liver enhances bile acid production, which facilitates fat absorption in the intestine and activates the Farnesoid X Receptor (FXR) in the liver and intestine to promote lipid assimilation [86]. This apparent paradox has been described as part of the Hinds’ hepatobiliary system [86]. The interaction between bilirubin and bile acids orchestrates contrasting mechanisms of fat burning and fat absorption, both of which are integral to hepatic physiology. They may not directly regulate each other, as bilirubin metabolism was not linked to changes in bile acid profiles in a human case–control study [87].

At excessively elevated levels (>400 μmol/L), bilirubin is recognized for its neurotoxic properties [88]. This phenomenon, frequently observed in infants and referred to as neonatal hyperbilirubinemia, occurs when the concentration of unconjugated bilirubin in the bloodstream exceeds the binding capacity of albumin, thereby permitting a greater amount of unconjugated bilirubin to traverse the blood–brain barrier [89]. The neurotoxicity may manifest through various mechanisms, including the absence of gut enzymes that metabolize bilirubin. In the brain, elevated doses of unconjugated bilirubin increase reactive oxygen species (ROS), endoplasmic reticulum stress, pro-inflammatory pathways, and glutamate excitotoxicity [90]. This is the opposite of what is observed for bilirubin effects on hepatocytes or immune cells. Nonetheless, the precise mechanisms underlying bilirubin neurotoxicity remain incompletely understood, and numerous questions persist.

Bilirubin, once considered solely as a toxic bile pigment, is now known to function as a significant antioxidant and hormone. Stocker et al. established in 1987 that bilirubin is a potent antioxidant [91]. Nevertheless, its antioxidant properties alone did not fully elucidate bilirubin’s influence on obesity and insulin resistance. In 2016, Stec et al. demonstrated that bilirubin also acts as a hormone by binding to the nuclear receptor PPARα at physiological concentrations [5]. Liver-specific PPARα knockout mice develop adiposity under normal conditions, leading to cardiovascular disease [92]. The interaction between bilirubin and PPARα recruits coactivators and upregulates the expression of PPARα target genes [3,52]. In cultured hepatocytes, PPARα is necessary for approximately 95% of the transcriptional response to bilirubin [4], suggesting that bilirubin primarily exerts its effects through PPARα, albeit likely not exclusively.

Hypobilirubinemia, characterized by low plasma bilirubin levels, might serve as a biomarker for adverse cardiometabolic outcomes [2]. Plasma bilirubin exhibits a negative correlation with indicators of adiposity and insulin resistance in humans [23]. A meta-analysis conducted by Yao et al. determined that elevated total bilirubin levels are associated with a 36% reduction in the incidence of first and recurrent myocardial infarctions [93]. Similar correlations have been identified with stroke, wherein increased bilirubin levels are associated with a decreased risk of stroke [94]; however, further research is required to elucidate the impact of bilirubin on stroke severity [95]. Additionally, circulating bilirubin levels are substantially lower in patients with rheumatic diseases compared to healthy controls, indicating diminished antioxidant and anti-inflammatory capacity [96]. Moreover, bilirubin exhibits a negative correlation with other diseases, including asthma [97], Crohn’s disease [98], and multiple sclerosis [99]. The inverse relationship between bilirubin levels and disease pathogenesis underscores the importance of further investigating its physiological functions.

The production of bilirubin by BVRA also indirectly modulates PPARα phosphorylation via glycogen synthase kinase-3β (GSK3β) [100]. BVRA functions not only as an enzyme responsible for bilirubin synthesis but also plays several crucial regulatory roles [86,101]. Specifically, BVRA serves as a kinase and scaffolding protein that governs insulin signaling pathways and can directly phosphorylate the insulin receptor [101]. Its kinase activity can target substrates at serine, threonine, or tyrosine residues, depending on the specific substrate [101]. Furthermore, BVRA operates as a transcription factor, controlling the expression of certain genes, including NRF2 [102]. Within gene promoters, BVRA binds hypoxia response elements (HREs) and antioxidant response elements (AREs), thereby regulating the transcription of target genes [55].

The function of BVRA has primarily been studied using knockout animal models. The global knockout of BVRA exacerbated diet-induced hepatic lipid accumulation and inflammation with no difference in body weight [103]. The liver-specific deletion of BVRA increased fasting glucose and insulin levels, potentially due to reduced hepatic insulin signaling and impaired glucose tolerance [100]. The loss of hepatic BVRA also induced liver steatosis and reduced phosphorylated GSK3β, thereby reducing hepatic glycogen storage [100]. Loss of BVRA also decreases serine 9 (S9) phosphorylation of GSK3β, thereby increasing its kinase activity [104], which directly phosphorylates PPARα at inhibitory serine 73 (S73), blocking its transcriptional activity [104]. The role of BVRA in suppressing lipid accumulation was validated in vitro utilizing murine liver and proximal tubule cells [105,106]. The loss of BVRA in adipose tissue led to increases in visceral fat, fasting blood glucose, and white adipose tissue inflammation and hypertrophy [107]. In humans, lower BVRA levels are associated with insulin resistance [103,108,109,110,111], diabetes, fatty liver disease, increased body mass, systolic blood pressure, immune regulation [112,113], and triglycerides [108,109,111]. Future studies are needed to investigate whether these protective effects of BVRA are mediated by its bilirubin production or by other functions.

Patients with Gilbert’s syndrome, caused by a polymorphism in the promoter of UGT1A1, have a reduction in UGT1A1 protein expression, leading to an increase in plasma bilirubin. Although Gilbert’s syndrome is typically asymptomatic, these patients have reduced risks of obesity [28], diabetes [114], and cardiovascular diseases [17]. The protective effect of Gilbert’s syndrome has been validated in a mouse model carrying the human Gilbert’s polymorphism, in which mice are protected from adiposity, insulin resistance, and diet-induced MASLD (Metabolic Dysfunction-Associated Steatotic Liver Disease) [115]. Plasma bilirubin is negatively correlated with adiposity, metabolic syndrome, and cardiovascular disease [2,20]. One hypothesis is that bilirubin metabolism and hepatic clearance are increased in individuals with metabolic syndrome. Hepatic UGT1A1 expression is elevated in obese mice and rats [116,117,118]. The hypothesis of increased bilirubin metabolism is supported by the positive correlation between plasma urobilin and markers of adiposity and insulin resistance in humans [23]. In mice with diet-induced obesity, inhibition of hepatic UGT1A1 with a liver-specific siRNA increased bilirubin and decreased plasma urobilin [119]. These mice exhibited reduced body fat percentage, improved glucose tolerance, and decreased hepatic lipid accumulation [119]. This suggests that targeting hepatic bilirubin metabolism through UGT1A1 may be a potential therapeutic approach to increase bilirubin and protect against MASLD and metabolic syndrome.

## 7. Therapeutic Potential of Modulating Bilirubin Transport and Clearance

Mild bilirubin accumulation in circulation has been associated with health benefits, suggesting that reducing bilirubin clearance and increasing its half-life may represent a novel treatment approach for metabolic diseases [21]. To achieve such accumulation, targeting key enzymes and transporters in bilirubin clearance, including UGT1A1, MRP2, OATP1B1, and OATP1B3, may offer promising strategies. In this section, we explore the potential of several pharmacological molecules that may promote mild bilirubin accumulation.

Atazanavir, a protease inhibitor approved by the U.S. Food and Drug Administration (FDA) for HIV/AIDS treatment, also acts as a competitive UGT1A1 inhibitor. It has been reported to increase plasma unconjugated bilirubin levels [120,121,122], suggesting that it may mildly increase bilirubin levels at low doses. Other antiretroviral drugs, such as indinavir, ritonavir, and nelfinavir, have also been reported to inhibit UGT1A1 [120,123,124]. However, further studies are needed to establish the dosage that can maintain bilirubin levels comparable to those in Gilbert syndrome, which are considered beneficial.

Inhibition of hepatic OATP1B1 and OATP1B3 can be an alternative strategy for mildly raising bilirubin levels. Atazanavir, nelfinavir, ritonavir, and indinavir, as well as troglitazone, are known inhibitors of OATP1B1 [120]; atazanavir also inhibits OATP1B3 [120]. Cyclosporine, an FDA-approved immunosuppressive substance, is a dual inhibitor of OATP1B1 and OATP1B3 [120] and has been shown to increase serum bilirubin levels [125]. Nonetheless, current evidence is insufficient to support the use of these drugs for sustained and protective bilirubin levels. Future studies are required to confirm their efficacy and safety.

MRP2 inhibition may also contribute to mildly increased bilirubin levels by impairing biliary excretion of bilirubin. Troglitazone has been reported to suppress MRP2 [120]; however, it has been withdrawn from the market due to liver toxicity. Other potential inhibitors of MRP2 include probenecid [126,127], cyclosporine [128,129], vindesine [130], and MK571 [131]. PSC833, a cyclosporine derivative, exhibits weak inhibition of MRP2 [132], which may mildly increase bilirubin levels. However, MRP2 not only facilitates the excretion of conjugated bilirubin via bile but also clears numerous metabolites from detoxification pathways; strong inhibition of MRP2 poses significant risks. Thus, developing and repurposing less potent derivatives, such as PSC833, may offer safer alternatives.

From a therapeutic perspective, low doses of certain UGT1A1, MRP2, OATP1B1, and OATP1B3 inhibitors may offer protective effects against cardiovascular and metabolic diseases by causing a mild elevation in serum bilirubin. However, inhibition of UGT1A1 and MRP2 may increase drug toxicity by reducing the clearance of other compounds metabolized via the same pathways. Hence, caution is warranted. In contrast, targeting OATP1B1 and OATP1B3 may pose a lower risk of interfering with detoxification. However, this still requires more supportive studies. Overall, while the modulation of these enzymes and transporters holds therapeutic potential, it is still too early to determine their clinical efficacy and safety. Further studies are essential to confirm the feasibility of these treatment strategies.

## 8. Bilirubin and Its Therapeutic Uses

Bilirubin has been studied as a therapeutic agent through the development of bilirubin nanoparticles. Multiple formulations of bilirubin nanoparticles have been synthesized; however, the most common are those created by covalently bonding bilirubin to polyethylene glycol to improve its solubility. Although the mechanisms governing the transport and metabolism of bilirubin nanoparticles remain inadequately understood, their administration every 48 h for four weeks in murine models has resulted in elevated plasma bilirubin levels [3,22,133]. Due to bilirubin’s historical perception as a toxic bile pigment, initial concerns were raised regarding its potential application in the treatment of hepatic dysfunction. Nonetheless, treatments with bilirubin nanoparticles in obese mice with MASLD and liver impairment have demonstrated improvements in hepatic function, with significant reductions in hepatic inflammation and the liver dysfunction biomarker aspartate transaminase (AST) levels [133]. Furthermore, these nanoparticles facilitate fat oxidation [133] and inhibit the accumulation of harmful ceramides within the liver [22]. Shinn et al. elucidated that bilirubin nanoparticles suppress hepatic fibrosis and impede the lipid-lowering effects of hepatic stellate cell (HSC) activation [134], thereby preventing the progression and development of liver fibrosis [134,135,136,137].

The finding that bilirubin can mitigate pathways of liver fibrosis is novel, particularly given that humans with cirrhosis typically exhibit elevated plasma bilirubin levels attributable to end-stage fibrosis [138]. These nanoparticles notably reduced body weight and increased lean muscle mass, indicating potential applications in obesity management [89]. The applications of bilirubin nanoparticles extend beyond MASLD and metabolic disorders [3]. In murine models, they have demonstrated protective effects against cardiac ischemia–reperfusion injury [139], suggesting their utility in treating cardiovascular diseases. Additionally, bilirubin nanoparticles have been shown to mitigate acute lung injury in septic mice [140] and to prevent graft-versus-host disease following transplantation [141]. Despite promising results from preclinical studies, further clinical trials are warranted to evaluate their efficacy in human patients, particularly regarding their potential effects on the Cardiovascular–Kidney–Metabolic (CKM) Syndrome, which has emerged as a significant global health concern [6].

## 9. Conclusions

Recent advances suggest that bilirubin functions as both a hormone and a regulator of metabolic homeostasis in humans and rodents. Following systemic circulation, bilirubin is primarily eliminated by the liver, secreted into bile, and excreted into the gastrointestinal tract via the biliary system. This process involves various transporters and enzymes, including OATP1B1, OATP1B3, UGT1A1, and MRP2, which facilitate bilirubin transport from the liver to the intestine. Beyond hepatic clearance, bilirubin’s influence extends, with modest increases in plasma levels associated with benefits such as reductions in fat-burning β-oxidation through its receptor, PPARα. Additionally, other proteins that bilirubin may interact with at jaundice levels warrant further investigation. In the intestine, the microbiota metabolizes bilirubin into urobilinogen, which is rapidly oxidized to urobilin. Notably, urobilin can be reabsorbed and may exert physiological effects distinct from those of bilirubin. It may contribute to obesity-related comorbidities such as MASLD, T2DM, and cardiovascular disease; however, further research is necessary to elucidate its precise effects. Although additional studies are required to confirm the physiological role of urobilin, the hypothesis that bilirubin and its metabolites have reciprocal regulatory effects on metabolism remains compelling. Since transporter- and enzyme-mediated pathways influence both plasma bilirubin levels and intestinal urobilin production, targeting these pathways could offer a novel therapeutic strategy for obesity, metabolic disorders, and related conditions. Nevertheless, as outlined, targeting these pathways could pose complications, and additional research is needed to understand how manipulating bilirubin transporter and reabsorption pathways affects human physiology and disease. Bilirubin nanoparticles show promise for managing cardiometabolic disorders, and determining whether their effects are altered by modification is crucial for advancing these compounds toward clinical application.

## Figures and Tables

**Table 1:** Bilirubin Isoforms.

Name	Solubility and Transport	Model	Ref.
**Bilirubin IXα**	Nearly insoluble Albumin as a carrier	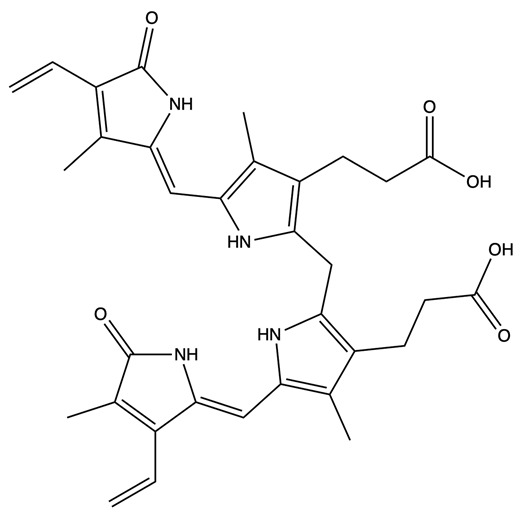	[59]
**Bilirubin IXα** (***ZE***)	Significantly higher solubility Albumin not required	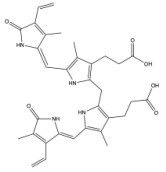	[60]
**Bilirubin IXα** (***EZ***)	Significantly higher solubility Albumin not required	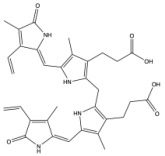	[60]
**Bilirubin IXα** (***EE***)	Significantly higher solubility Albumin not required	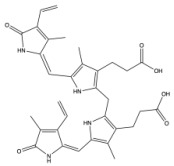	[60]
**Bilirubin XIIIα**	Nearly insoluble Not commonly found in the human body	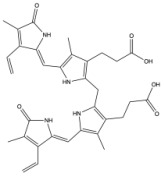	[59,61]
**Bilirubin IIIα**	Nearly insoluble Not commonly found in the human body	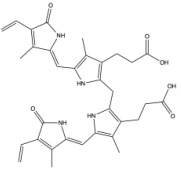	[59]

**Table 2:** The expression of bilirubin-metabolizing enzymes in the four main gut bacteria phyla.

Phylum	Produce BilR	Produce GUS
Bacteroidetes	✗	✓
Firmicutes	✓	✓
Proteobacteria	Some (Flavobacteria)	✓
Actinobacteria	Some (Bifidobacterium)	✓

**Table 3:** The Properties of Bilirubin Intestinal Metabolites and the Related Bacterial Enzymes.

Name	Properties	Clinical Relevance
Unconjugated Bilirubin	- Water-insoluble. - Inhibits proteases.	- Mildly elevated levels are associated with improved metabolic outcomes. - May have therapeutic potential for IBS, insulin-resistant diabetes, and cardiovascular and metabolic disorders.
Conjugated Bilirubin	- Water-soluble. - Transported in bile from the liver to the intestines. - Antioxidant in serum.	- Highly elevated levels can indicate liver disease or biliary obstruction.
GUS Enzymes	- Deconjugate conjugated bilirubin. - Found in intestinal microbiota, for example, the Alistipes genus.	- Activity is lower in high-proteolytic-activity IBS.
BilR Enzyme	- Reduces the double bonds to form urobilinogen. - Found in intestinal microbiota, mainly those of the Clostridium genus.	- Lack of BilR may be associated with neonatal jaundice. - Found in nearly all people other than infants and those with inflammatory bowel disease.
Urobilinogen	- Water-soluble. - Mostly remains in the intestines and is further metabolized. - Antioxidants in the intestines.	- High serum and urine levels can suggest liver disease or hemolytic anemia.
Urobilin	- Water-soluble. - Greater than 50% can be reabsorbed by the hepatic portal vein.	- High blood levels can indicate metabolic dysfunction such as insulin-resistance, type 2 diabetes mellitus, or cardiovascular diseases

## Data Availability

This review article contains no datasets generated or analyzed during the current study.
